# Estrogen Receptor α Participates to the Beneficial Effect of Red Wine Polyphenols in a Mouse Model of Obesity-Related Disorders

**DOI:** 10.3389/fphar.2016.00529

**Published:** 2017-01-10

**Authors:** Daniela Leonetti, Raffaella Soleti, Nicolas Clere, Luisa Vergori, Caroline Jacques, Lucie Duluc, Catherine Dourguia, Maria C. Martínez, Ramaroson Andriantsitohaina

**Affiliations:** ^1^INSERM U1063, Stress Oxydant et Pathologies Métaboliques, Université d’AngersAngers, France; ^2^Centre Hospitalier Universitaire d’AngersAngers, France

**Keywords:** estrogen receptor α, metabolic disorders, obesity, polyphenols, vascular disorders

## Abstract

Red wine polyphenol extracts (polyphenols) ameliorate cardiovascular and metabolic disorders associated with obesity. Previously, we demonstrated that the alpha isoform of estrogen receptor (ERα) triggers the vascular protection of polyphenols. Here, we investigated the contribution of ERα on the effects of polyphenols on cardiovascular and metabolic alterations associated with obesity. We used ovariectomized wild type or ERα-deficient mice receiving standard (SD) or western (WD) diets, or SD and WD containing polyphenols (SD+polyphenols and WD+polyphenols, respectively) over a 12-week period. Body weight was measured during treatment. Echocardiography examination was performed before sacrifice. Blood and tissues were sampled for biochemical and functional analysis with respect to nitric oxide (NO^•^) and oxidative stress. Vascular reactivity and liver mitochondrial complexes were analyzed. In WD-fed mice, polyphenols reduced adiposity, plasma triglycerides and oxidative stress in aorta, heart, adipose and liver tissues and enhanced NO^•^ production in aorta and liver. ERα deletion prevented or reduced the beneficial effects of polyphenols, especially visceral adiposity, aortic and liver oxidative stresses and NO^•^ bioavailability. ERα deletion, however, had no effect on polyphenol’s ability to decrease the fat accumulation and oxidative stress of subcutaneous adipose tissue. Also, ERα deletion did not modify the decrease of ROS levels induced by polyphenols treatment in the visceral adipose tissue and heart from WD-fed mice. Dietary supplementation of polyphenols remarkably attenuates features of metabolic syndrome; these effects are partially mediated by ERα-dependent mechanisms. This study demonstrates the therapeutic potential of this extract in metabolic and cardiovascular alterations linked to excessive energy intake.

## Introduction

Obesity is defined as abnormal or excessive fat accumulation associated with an increased risk of premature death due to an increased incidence of metabolic diseases such as insulin resistance, type 2 diabetes, dyslipidemia, non-alcoholic fatty liver disease and coronary heart disease. Based on epidemiological studies reporting a greater reduction in cardiovascular risk and metabolic disorders associated with polyphenol-rich diet, several dietary and pharmacological approaches have been proposed to manage or prevent obesity and obesity-related diseases. Polyphenols are bioactive food compounds primarily present in fruits and vegetables and exert health benefits protecting against metabolic and cardiovascular disturbances (Andriantsitohaina et al., 2012). Polyphenols, in particular those derived from red wine, possess anti-aggregatory platelet activity, antioxidant and free radical scavenging properties (Curin et al., 2006; Pechanova et al., 2006), as well as lipid- and lipoprotein-lowering effects (Curin et al., 2006). We have reported that red wine polyphenols (polyphenols) are powerful vasodilators through the generation of NO^•^ and can act on the expression of protective genes of the cardiovascular system (Andriambeloson et al., 1997). Importantly, in a rat model of insulin-resistance, polyphenols prevent hypertension, cardiac hypertrophy as well as ROS production in aorta and heart (Bernátová et al., 2002; Al-Awwadi et al., 2005). Moreover, in a context of obesity, polyphenols ameliorate glucose and lipid metabolism, cardiac function, decrease peripheral resistance and improve endothelium-dependent relaxation by enhancing NO^•^ bioavailability (Agouni et al., 2009).

Among the different classes of polyphenolic compounds present in red wine, anthocyanins and oligomeric condensed tannins exhibit pharmacological profiles comparable with total extracts in terms of endothelial-dependent NO^•^-mediated vasodilatation (Andriambeloson et al., 1998). Among anthocyanins identified in wine, only delphinidin causes endothelium-dependent relaxation, however, it is slightly less potent than total extract (Andriambeloson et al., 1998).

A growing body of evidence demonstrates that estrogenic signaling has an important role in obesity development (Musatov et al., 2007; Xu et al., 2011). Indeed, 17 β-estradiol acts on the α isoform of receptor (ERα) and protects against obesity-related diseases (Musatov et al., 2007; Xu et al., 2011). Of note, ERα has been identified as one of the key receptors transducing vascular effects exerted by polyphenols, particularly for delphinidin (Chalopin et al., 2010). These compounds interact directly with ERα, activating molecular pathways including Src, ERK1/2, eNOS, leading to endothelial NO^•^ production, and consequently vasorelaxation (Chalopin et al., 2010).

Based on these observations, the present study was designed to investigate the contribution of ERα to the effect of polyphenols in an experimental model of obesity and related metabolic disorders using ERα WT or KO mice. In order to avoid the cofounding effect of circulating estrogens on the effect of polyphenols, known to possess structural similarities with estrogens (Bowers et al., 2000; Chalopin et al., 2010), mice were OVX. Animals were fed with a SD or a WD or SD and WD containing polyphenols (SD+polyphenols and WD+polyphenols, respectively). Particular emphasis has been placed on body weight and fat deposition, glucose and lipid metabolism, cardiac and vascular functions, tissue NO^•^ and ROS generation and hepatic mitochondrial function.

## Materials and Methods

### Products

Polyphenols (exGrape^®^TOTAL) were obtained from Grap’Sud (Cruviers-Lascours, France). The phenolic composition of dry powder in mg/g was: 2.72 hydroxybenzoic acids (gallic acid and ethyl gallate), 315.60 flavan-3-ols (catechins, epicatechins, B1, B2, B3, B4, galloyl, (epi)cat-epigallocatechin dimers, trimer), 0.81 stilbenes (trans-resveratrol, 𝜀-viniferin, cis-resveratrol), 0.58 mg/g dihydroflavonol (astilbin), 3.62 hydroxycinnamic acids (caftaric acid, coutaric acid, caffeic acid, fertaric acid, cumaric acid), 15.10 flavonols (myricetin 3-*O*-glucuronide, myricetin 3-*O*-glucoside, quercetin 3-*O*-galactoside, quercetin 3-*O*-glucuronide, quercetin 3-*O*-glucoside, laricitrin 3-*O*-glucoside, myricetin aglycone, syringetin glucoside, quercetin aglycone), 3.89 anthocyanins [(epi)cat-malvidin 3-*O*-glucoside, delphinidin 3-*O* glucoside, cyanidin 3-*O*-glucoside, petunidin 3-*O*-glucoside, (malvidin + peonidin] 3-*O*-glucoside, pyranomalvidin pyruvic acid, malv-ethyl-(epi)catechin, (malvidin + peonidin) acetyl glucoside, pyranomalvidin 3-*O*-coumaroyl glucoside, petunidin 3-*O*-coumaroyl glucoside, (malvidin + peonidin) 3-*O*-coumaroyl glucoside, pyranomalvidin vinyl catechol, pyranomalvidin vinyl phenol).

Polyphenols were mixed into a chow supplied in powder form at 150 mg/kg. By taking into account that the weight of the mouse is, in average, 22 g (please **Table 1**) and that each mouse eats 4 g of chow per day, the mouse consumes 0.6 mg of polyphenols per day. Thus, the mouse received a dose of polyphenols of 27.3 mg/kg/day. This dose is consistent with our former studies (Diebolt et al., 2001; Baron-Menguy et al., 2007; Agouni et al., 2009; Chalopin et al., 2010, 2014) and is comparable to the concentration that produced maximal relaxation of mouse aortic rings *ex vivo*. This was also compatible with a human consumption of one to two glasses of red wine per day and was in the range of that shown to possess beneficial effects on oxidative damage in humans (Covas et al., 2010).

**TABLE 1:** Animal characteristics following 12 weeks of diets.

	WT				KO				
	*SD*	*SD* + Polyphenols	WD	WD + Polyphenols	*SD*	*SD*+Polyphenols	WD	WD+Polyphenols
Initial body weight (bw, g)	19:8 ± 0:4	19:5 ± 0:6	20:2 ± 0:6	19:3 ± 0:3	20:1 ± 1:1	19:6 ± 0:7	17:7 ± 1:1	20:3 ± 0:5
Final body weight (g)	20:9 ± 0:6	20:1 ± 0:4	26 ± 0:5^∗∗∗^	23:5 ± 0:5^§§^	20:5 ± 0:9	19:6 ± 0:5.	23:0 ± 1:6	27:6 ± 0:8^¥¥¤¤¤¤^
Liver weight (bw%)	6:0 ± 0:3	5:4 ± 0:5	6:3 ± 0:3	6:0 ± 0:2	4:6 ± 0:2^∗∗^	4:8 ± 0:2	4:3 ± 0:2^##^	4:5 ± 0:2
Heart (bw%)	0:7 ± 0:03	0:7 ± 0:07	0:5 ± 0:03	0:6 ± 0:07	0:5 ± 0:05	0:5 ± 0:05	0:5 ± 0:03	0:4 ± 0:02
Visceral adipose tissue (bw%)	3:6 ± 0:3	3:3 ± 0:5	8:5 ± 1:3^∗∗∗^	4:4 ± 0:5^###^	2:8 ± 0:3	2:9 ± 0:3	5:6 ± 1:1	7:5 ± 0:9^¥¤¤¤^
Subcutaneous adipose tissue (bw%)	1:7 ± 0:3	1:1 ± 0:2	3:9 ± 0:2^∗∗^	2:4 ± 0:4	1:9 ± 0:2	2:2 ± 0:2	5:0 ± 0:5^††††^	5:6 ± 0:7^¤¤¤¤¥¥¥¥^
Adiposity (bw%)	5:3 ± 0:2	4:4 ± 0:5	12:4 ± 1:4^∗∗∗∗^	6:8 ± 0:7^###^	4:7 ± 0:5	5:2 ± 0:5	10:6 ± 1:5^††^	13:1 ± 1:6^¤¤¤¤¥¥¥^

*The table shows the weight of mice, heart, visceral and subcutaneous adipose tissue and liver of ERα WT or KO mice fed with SD, WD, SD + Polyphenols or WD + Polyphenols for 12 weeks. The data are given as the mean ± SEM. Statistical analyses were performed by one-way ANOVA and Holm–Sidak’s post hoc test, ^∗∗^P < 0.01, ^∗∗∗^P < 0.001, ^∗∗∗∗^P < 0.0001, vs. WT SD group; §§P < 0.01 vs. WT SD + polyphenols group; ¥ < 0.05, ¥¥ < 0.0 vs. KO WD group; ¤¤¤P < 0.001, ¤¤¤¤P < 0.0001 vs. KO SD + polyphenols group; ##P < 0.01; ###P < 0.001 vs. WT WD group; ††P < 0.01, †††P < 0.0001 vs. KO SD group.*

SD (U8960P version 0066) and WD (U8958P version 0052) as well as those containing polyphenols were obtained from SAFE (Augy, France) (Supplemental Table 1).

SD contains 5% of fat based on its composition in g/kg which represents 12% calories of total diet. With regard to WD, it contains 21.5% of fat in g/kg that corresponds to 36.8% calories.

### Ethics Statement

The local ethics committee (“Comité d’éthique en expérimentation animale Pays de la Loire”) approved the animal protocol followed in the present study (CEEA.2011.40). This study was carried out in strict accordance with the guidelines and authorization with French Ministry of Agriculture regulations based on European Community.

### Animals and Protocol Design

Eight-week-old female C57bl/6 ERα WT or KO mice were purchased from the Jackson Laboratories (Bar Harbor, ME) and maintained at 23 ± 2°C under a 12 h light-dark cycle. Mice were OVX following a week of acclimation.

Mice were anesthetized using isoflurane (5% in induction chamber, 2% in mask, flow of oxygen 1 L/minute). As an analgesic, buprenorphine (0.1 mg/kg sc injection) was administered pre- and post-surgery. During and after surgery, mice were placed on heating pad.

After 7 days, both strains were randomly divided into four groups SD, WD, SD + polyphenols and WD + polyphenols during 12 weeks (*n* = 7/group). Mice were allowed *ad libitum* access to water and diets. Animal body weight was measured weekly. One week before sacrifice, mice were anesthetized for echocardiography examination. After treatment, mice were euthanized by gradient CO_2_ inhalation. First, we evaluated the efficiency of ovariectomy assessed by uterus atrophy. The comparison of uterus weight of OVX mice with uterus weight of non-OVX mice was performed. Only the animals showing uterus atrophy have been included in the study which explain why the final number of animals was different in each group (WT SD *n* = 5, WT SD +polyphenols *n* = 6, WT WD *n* = 5, WT WD + polyphenols *n* = 7, KO SD *n* = 5, KO SD + polyphenols *n* = 5, KO WD *n* = 4, KO WD + polyphenols *n* = 5). Then, blood, tissues and organs were sampled for biochemical and/or histological analysis.

### Biochemical Analyses

Blood was collected by cardiac puncture at sacrifice and blood was centrifuged for 15 min at 950 *g* and room temperature to obtain plasma. Fasting glucose, triglycerides, total cholesterol, LDL-cholesterol and HDL-cholesterol were measured using Konelab^TM^ 20 Clinical Chemistry Analyzer (Thermo Scientific, Waltham, MA, USA).

### *Ex vivo* Vascular Reactivity

Mice aorta was dissected and placed in modified Krebs–Henseleit bicarbonate solution. Aortic rings were then mounted on a wire myograph (Danish MyoTechnology, Aarhus, Denmark) and filled with PSS, as previously described Chalopin et al. (2010). Endothelium-dependent vasodilatation in response to Ach (1 nmol/L to 10 mmol/L; Sigma–Aldrich, St. Quentin, Fallavier, France) was studied in aortic rings with functional endothelium pre-contracted with the thromboxane A2 agonist (9,11-dideoxy-11a, 9a-epoxymethanoprostaglandin F2-a, U46619) (Sigma–Aldrich) at 80% of their maximal response. Then, concentration-response curves were constructed by cumulative application of 5-HT (1 nmol/L to 10 μmol/L; Sigma–Aldrich) to vessels with functional endothelium.

### Echocardiography Examination

*In vivo* transthoracic echocardiography was performed using a VEVO 770 ultrasound machine from FUJIFILM Visualsonics (Toronto, ON, Canada) equipped with a 30-MHz imaging transducer in mice anesthetized with isoflurane. Briefly, a two-dimensional short axis view of the left ventricle was obtained in order to record M-mode tracings. LVEDD, LVSED and COI were evaluated.

### Nitric Oxide (NO^•^) and Reactive Oxygen Species (ROS) Determination by Electron Paramagnetic Resonance (EPR)

Measurements were performed on a table-top x-band spectrometer Miniscope (Magnettech, MS200, Berlin, Germany). Detection of NO^•^ production in aorta, visceral and subcutaneous adipose tissues, heart and liver was performed using DETC (Sigma–Aldrich) as a spin trap, whereas ROS detection was performed using deferoxamine-chelated Krebs–Hepes solution containing CMH (500 μmol/L, Noxygen, Mainz, Germany), deferoxamine (25 μmol/L, Sigma–Aldrich), and DETC (5 μmol/L) (Agouni et al., 2009). Signal values are expressed in A.U. as data was corrected for total amplitude/mg weight of dried tissue, as previously described Agouni et al. (2009).

### Mitochondrial Enzyme Activities in Liver

Activities of CS, complex I, II, and IV of mitochondria were spectrophotometrically measured on liver homogenates at 37°C and expressed in nmol of product formed per minute and per mg of protein in homogenates, as previously described Mingorance et al. (2012).

### Statistical Analysis

Data were expressed as mean ± SEM and *n* represents the number of animals. The statistical software GraphPad Prism 7.0 (GraphPad Software Inc., San Diego, CA, USA) was used for data analysis. The difference between groups was performed by repeated measures two-way ANOVA for the evolution of body weight gain. For all the other experiments one-way ANOVA was applied. When ANOVA demonstrated a significant interaction between variables, *post hoc* analyses were performed by the multiple-comparison Holm–Sidak’s test. *P* < 0.05 was considered to be statistically significant.

## Results

### Effect of Polyphenols on Body and Organ Weight

**FIGURE 1:**
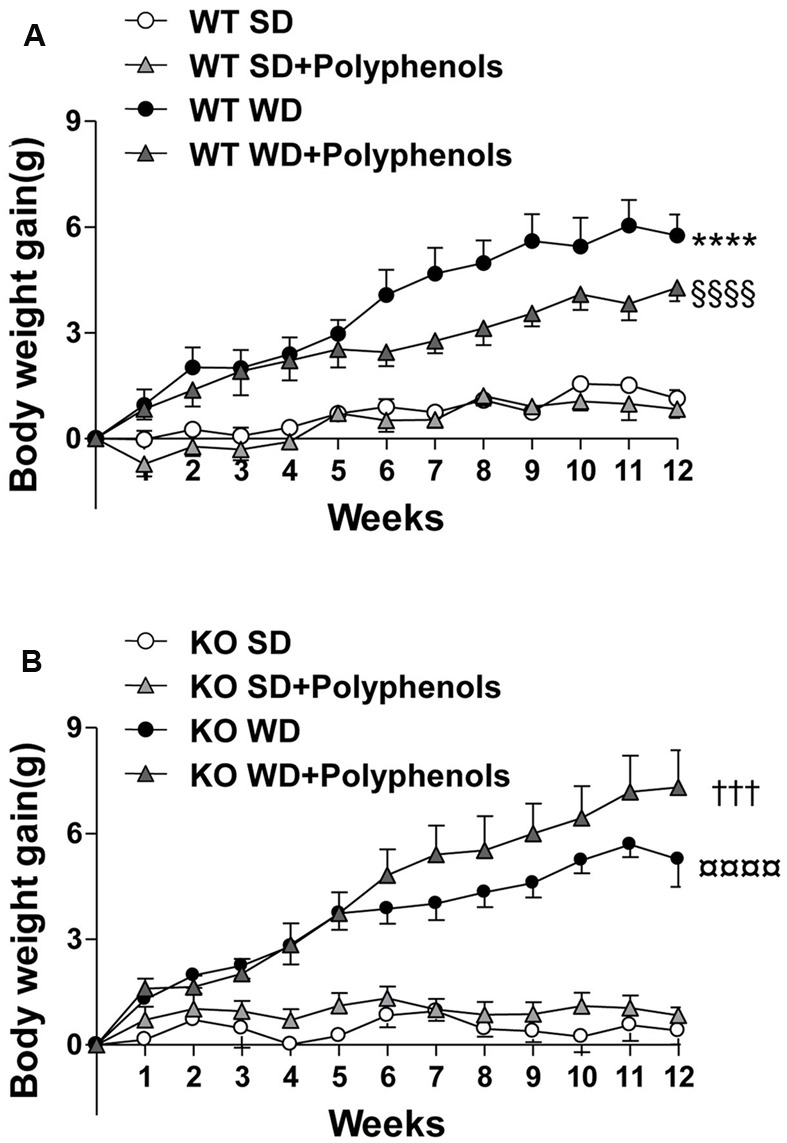
**The evolution of (A,B)** body weight gain of ERα WT **(A)** and KO **(B)** mice receiving normal diet (SD), western diet (WD), or SD and WD containing polyphenols (SD + polyphenols and WD + polyphenols, respectively) during 12 weeks. The body weight was recorded twice a week. The data are expressed as the mean ± SEM. Statistical analyses were performed by repeated measures two-way ANOVA and Holm–Sidak’s *post hoc* test, ^∗∗∗∗^*P* < 0.0001 vs. WT SD, §§§§*P* < 0.0001vs. WT SD + RWP, †††*P* < 0.001 vs. KO SD, *P* < 0.0001 vs. KO SD + RWP.

WD diet induced a time-dependent increase in body weight gain in WT mice compared to WT SD-fed mice (*P* < 0.0001) (**Figure 1**). Polyphenols did not modify body weight of WT mice fed with SD (**Table 1**) and it did not significantly reduce the body weight gain induced by WD compared to control. In KO mice, WD increased body weight gain (*P* < 0.0001) that was not significantly reduced upon polyphenol treatment.

In WT mice fed with WD, the increase of body weight was associated with higher visceral and subcutaneous adiposity compared to WT SD mice (*P* = 0.0002 and *P* = 0.0045, respectively) (**Table 1**). Interestingly, polyphenols decreased visceral (*P* = 0.0022) but not subcutaneous adiposity compared to WT WD mice. In mice lacking ERα, WD augmented subcutaneous adipose tissue weight (*P* < 0.0001). Polyphenols failed to modify the increase in weight of adipose tissues in mice lacking ERα treated with WD (**Table 1**).

Liver and heart weight was not modified upon WD and polyphenols supplementation in WT mice compared to SD-fed mice. Deletion of ERα decreased liver weight in mice fed with SD and WD (*P* = 0.0053 and *P* = 0.0041, respectively) compared to WT groups. Polyphenols had no effect on both liver and heart weight.

Altogether, these data show that the ability of polyphenols to reduce adiposity requires the presence of ERα.

### Effect of Polyphenols on Biochemical Plasma Parameters

**FIGURE 2:**
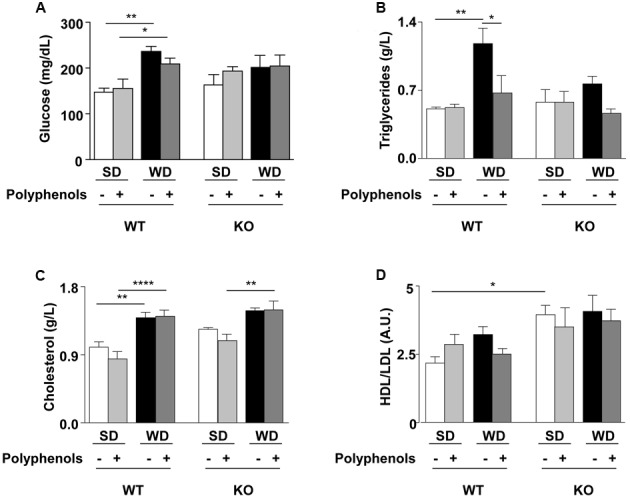
**(A)** Glucose, **(B)** triglycerides, **(C)** cholesterol plasma levels and **(D)** HDL/LDL ratio of ERα WT and KO mice receiving normal diet (SD), western diet (WD), or SD and WD containing polyphenols (SD + polyphenols and WD + polyphenols, respectively) during 12 weeks. The data are given as the mean ± SEM. Statistical analyses were performed by one-way ANOVA and Holm–Sidak’s *post hoc* test, ^∗^*P* < 0.05, ^∗∗^*P* < 0.01, ^∗∗∗∗^*P* < 0.0001.

Plasma levels of glucose, triglycerides and cholesterol were greater in WT mice fed with WD compared to WT SD mice (*P* = 0.0025, *P* = 0.0017 and *P* = 0.00781, respectively) (**Figure 2**). HDL/LDL ratio (**Figure 2**) and insulin levels (1.7 ± 0.3 vs. 1.8 ± 0.6 ng/dL) were not different in WT SD and WT WD mice.

Polyphenols normalized circulating levels of triglycerides (*P* = 0.0115) without affecting glycemia, cholesterolemia and HDL/LDL ratio in WT mice fed with WD.

Deletion of ERα increased HDL/LDL ratio in SD-fed mice compared to WT SD group (*P* = 0.0086), without modifications of other evaluated parameters.

WD significantly enhanced cholesterol levels in KO mice fed with polyphenols compared to those fed with SD plus polyphenols (*P* = 0.0202). Diet supplementation with polyphenols did not modify circulating parameters in KO mice.

Thus, ERα was involved in the WD-induced hyperglycemia and hypertriglyceridemia and polyphenols did not further modify these parameters.

### Effects of Polyphenols on Aorta

**FIGURE 3:**
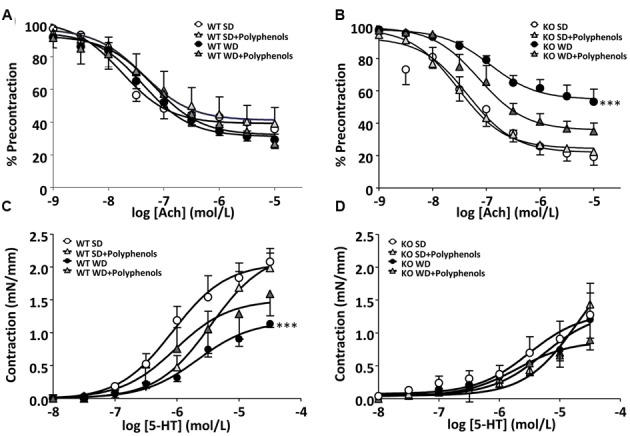
**Concentration-response curves to (A,B)** Ach and **(C,D)** 5-HT in aortic rings from ERα WT **(A,C)** and KO **(B,D)** mice receiving normal diet (SD), western diet (WD), or SD and WD containing polyphenols (SD + polyphenols and WD + polyphenols, respectively) during 12 weeks. The data are expressed as the mean ± SEM. Statistical analyses were performed by one-way ANOVA and Holm–Sidak’s *post hoc* test, ^∗∗∗^*P* < 0.001.

Both WD and polyphenols did not modify the endothelium-dependent relaxation to Ach in aortic rings from WT mice (**Figure 3A**). Deletion of ERα did not affect relaxation to Ach compared WT mice (**Figure 3B**). In aortas from ERα KO mice, WD significantly reduced maximal response to Ach when compared to that from KO SD-fed mice (*P* = 0.0002). Polyphenols treatment did not significantly affect relaxation to Ach in aorta from both SD and WD-treated mice.

5-HT induced a concentration-dependent contraction in aortic rings from all groups of mice. 5-HT response was significantly decreased in mice receiving WD compared to those receiving SD (*P* = 0.0001) (**Figure 3C**). Polyphenols failed to improve the contractile response to 5-HT in WT mice independently of diet. Although not statistically significant, in the aortic rings from KO mice, the maximal contraction induced by 5-HT was lower than those of WT mice. Neither WD nor polyphenols modified 5-HT-evoked responses in aorta for ERα-deficient mice (**Figure 3D**).

### Effects of Polyphenols on Heart

Echocardiographic analysis showed no significant modifications in heart structure and function, whatever the mouse strains and diets (**Table 2**).

**Table 2:** Cardiac function following 12 weeks of diets.

	WT				KO			
	*SD*	*SD* + Polyphenols	WD	WD + Polyphenols	*SD*	*SD* + Polyphenols	WD	WD + Polyphenols
LVEDD (mm)	3.5 ± 0.2	3.4 ± 0.2	3.7 ± 0.2	3.6 ± 0.1	3.4 ± 0.1	3.3 ± 0.1	3.2 ± 0.1	3.6 ± 0.2
LVESD (mm)	2.2 ± 0,1	2.2 ± 0.2	2.6 ± 0.2	2.3 ± 0.1	2.1 ± 0.1	2.4 ± 0.1	1.8 ± 0.1	2.6 ± 0.2
EF (%)	69.1 ± 2.8	70.9 ± 3.6	69.6 ± 2.8	66.4 ± 2.2	70.4 ± 1.1	60.8 ± 2.2	74.7 ± 2.3	65.1 ± 4.4
COI (mL/min/g)	0.8 ± 0.05	0.7 ± 0.07	0.7 ± 0.1	0.7 ± 0.05	0.8 ± 0.1	0.6 ± 0.1	0.8 ± 0.2	0.6 ± 0.03

*The table shows the LVEDD, LVEDD, EF, COI in ERα WT or KO mice fed with SD, WD, SD + Polyphenols or WD + Polyphenols for 12 weeks. The data are given as the mean ± SEM.*

### Effect of Polyphenols on Liver

**FIGURE 4:**
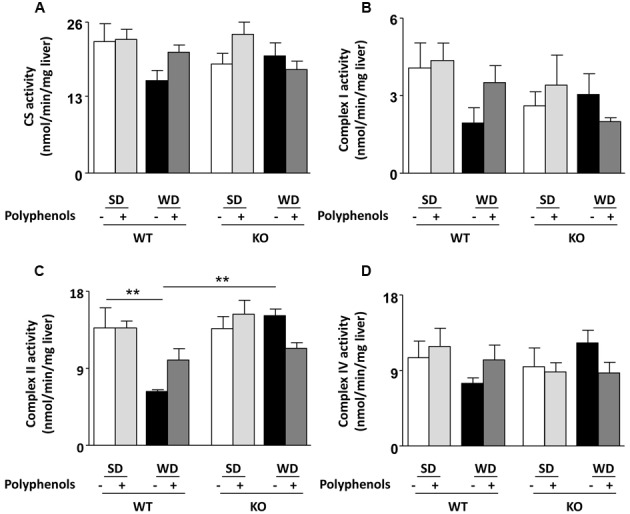
**Measurement of (A–D)** enzymatic activities of mitochondrial respiratory chain complexes (CS, I, II, IV) in liver from ERα WT and KO mice receiving normal diet (SD), western diet (WD), or SD and WD containing polyphenols (SD + polyphenols and WD + polyphenols, respectively) during 12 weeks. The data are expressed as the mean ± SEM. Statistical analyses were performed by one-way ANOVA and Holm–Sidak’s *post hoc* test, **P < 0.01.

In WT mice, among all liver enzyme activities tested, WD only reduced complex II activity (*P* = 0.0092) (**Figure 4C**). Neither polyphenols nor deletion of ERα had any effect on enzyme activity in SD-fed mice (**Figures 4A,B,D**). Complex II activity was greater in KO compared to WT mice fed with WD (*P* = 0.0026).

### Effect of Polyphenols on NO^•^ Production

**FIGURE 5:**
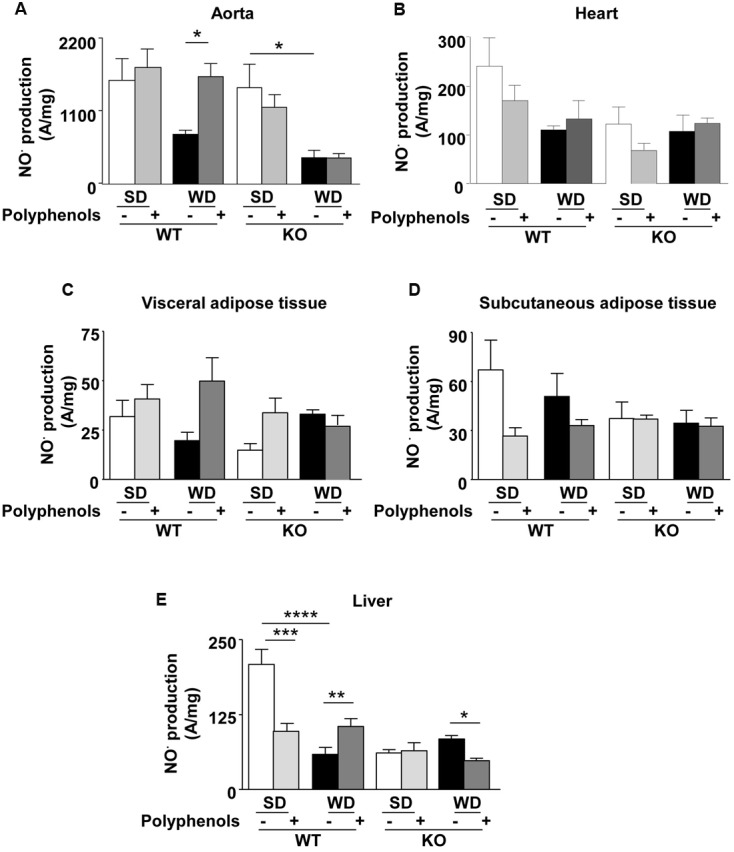
**Measurement of *in situ* NO^•^ productions by electron paramagnetic resonance in aorta (A)**, heart **(B)**, visceral **(C)** and subcutaneous **(D)** adipose tissue, and liver **(E)** from ERα WT and KO mice receiving normal diet (SD), western diet (WD), or SD and WD containing polyphenols during 12 weeks. The data are expressed as the mean ± SEM. Statistical analyses were performed by one-way ANOVA and Holm–Sidak’s *post hoc* test, ^∗^*P* < 0.05, ^∗∗^*P* < 0.01, ^∗∗∗^*P* < 0.001.

Aorta from WT mice fed with WD displayed a decrease in NO^•^ production which was not significantly different from that observed in the SD group (**Figure 5A**). Polyphenols caused a significant (*P* = 0.00105) increase in NO^•^ production in WT animals fed a WD, but not in those fed a SD. NO^•^ production in ERα-deficient mice fed with WD was lower than those of SD group (*P* = 0.0177) (**Figure 5A**). Polyphenols did not modify NO^•^ production in aortas taken from KO mice fed with both diets. These data provide evidence that ERα is involved in the increase of NO^•^ production induced by polyphenols.

In the heart, although not statistically significant, EPR measurement revealed a reduction of 45% in NO^•^ production in WT mice fed with WD. Polyphenols did not significantly modify NO^•^ production in WT in both SD and WD mice. In ERα-deficient mice, NO^•^ production did not change independent of diet and polyphenol supplementation (**Figure 5B**).

NO^•^ production in both visceral and subcutaneous adipose tissue was not significantly different, independent of both diets and strains (**Figures 5C,D**).

In WT, WD significantly reduced hepatic NO^•^ production (71%) compared to SD-fed mice (*P* < 0.00001) (**Figure 5E**). Polyphenols reduced NO^•^ levels (52%) in SD-fed mice (*P* = 0.0006); however, it enhanced NO^•^ production (42%) in WD-fed mice (*P* = 0.0303). Deletion of ERα profoundly decreased NO^•^ production (71%) in SD-fed mice (*P* < 0.00001). In ERα deficient mice, WD did not significantly modify NO^•^ production compared to SD-fed mice. In WD-fed ERα deficient mice, supplementation of polyphenols further reduced NO^•^ production (∼40%) (*P* = 0.0238). These results suggest that polyphenols enhanced NO^•^ bioavailability in the liver of WD-fed mice through an ERα-dependent mechanism.

### Effect of Polyphenols on ROS Production

**FIGURE 6:**
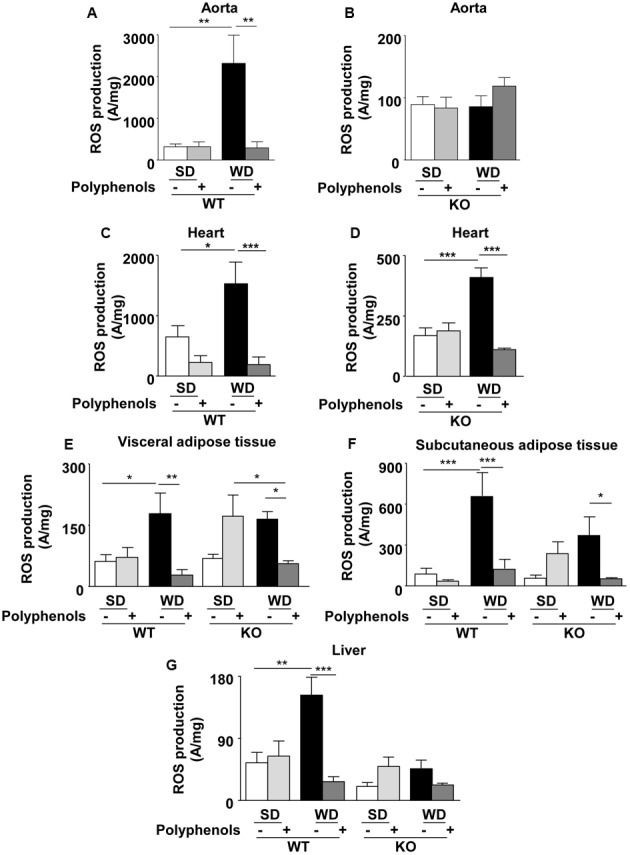
**Measurement of *in situ* ROS productions by electron paramagnetic resonance in aorta (A,B)**, heart **(C,D)**, visceral **(E)** and subcutaneous **(F)** adipose tissue, and liver **(G)** from ERα WT and KO mice receiving normal diet (SD), western diet (WD), or SD and WD containing polyphenols during 12 weeks. The data are expressed as the mean ± SEM. Statistical analyses were performed by one-way ANOVA and Holm–Sidak’s *post hoc* test, ^∗^*P* < 0.05, ^∗∗^*P* < 0.01, ^∗∗∗^*P* < 0.001.

In WT mice, ROS production was greater in aortas taken from WD-fed mice compared to SD group (*P* = 0.0001) (**Figure 6A**). Polyphenols did not modify oxidative stress in WT SD mice, but it completely prevented the increase of ROS induced by WD (*P* = 0.001). In KO mice, no changes in ROS production were observed (**Figure 6B**). These data suggest that the polyphenol-induced prevention of oxidative stress in aorta was dependent on ERα.

Reactive oxygen species production in the heart of WT mice fed with WD was greater than in SD mice (*P* = 0.0351) (**Figure 6C**). Interestingly, polyphenols decreased oxidative stress in WD-fed mice (87%) (*P* = 0.0005). WD increased ROS production in hearts from ERα-deficient mice (**Figure 6D**) (*P* = 0.0005). Polyphenols was still effective in reducing ROS production by 73% in hearts taken from ERα-deficient mice fed with WD (*P* = 0.0001). These data suggest that polyphenols exerts a protective effect against oxidative stress in the heart independently of the presence of ERα.

Reactive oxygen species production was greater in visceral adipose tissue taken from WD-fed mice compared to SD in WT mice (*P* = 0.04) (**Figure 6E**). Polyphenols did not modify ROS levels in WT SD mice; however, it completely prevented oxidative stress induced by WD (68.5%) (*P* = 0.0029) (**Figure 6E**). In KO mice, WD did not significantly increase ROS production compared to SD. Polyphenols tended to enhance oxidative stress in SD mice (62.5%), but it significantly (*P* = 0.0471) decreased ROS levels in WD-fed mice (65.8%). These results suggest that in visceral adipose tissue polyphenols reduced oxidative stress independently of ERα.

In WT mice, WD significantly (*P* = 0.0007) increased ROS production in subcutaneous adipose tissue (86.7%) mice compared to SD mice (**Figure 6F**). ROS level was significantly (*P* = 0.0007) reduced upon polyphenol treatment (83%). In KO mice, polyphenols completely prevented (82.3%) WD-induced ROS production (*P* = 0.0463). These data suggest that polyphenols prevented oxidative stress in subcutaneous adipose tissue independently of ERα.

In WT mice, liver ROS production was greater (64%) in those fed with WD compared to SD (*P* = 0.0094) (**Figure 6G**). Polyphenols had no effect on SD-fed WT mice; however, it significantly reduced oxidative stress (82%) in WD-fed WT animals (*P* = 0.0006). ROS levels were not significantly different in WT and KO mice fed with SD. Polyphenols did not affect ROS production in both SD-fed and WD-fed KO mice. These data suggest that polyphenols prevented oxidative stress in liver dependently of ERα.

## Discussion

This study provides new and interesting evidence for the beneficial effects of polyphenols on metabolic and cardiovascular disorders induced by WD. First, polyphenols significantly improved features of metabolic disorders induced by WD including reduction of visceral and subcutaneous adipose tissue accumulation and plasma triglyceride levels in WT mice. Second, polyphenols increased NO^•^ bioavailability resulting from both enhanced NO^•^ production and decreased ROS levels i n the aorta and liver. Third, polyphenols decreased oxidative stress in visceral and subcutaneous adipose tissues and heart. Fourth, deletion of ERα prevented the beneficial effects induced by supplementation with polyphenols in WD-fed mice. Indeed, we clearly demonstrated that the reduction of adiposity, and NO^•^ bioavailability, as well as, the improved activity of some hepatic mitochondrial complexes are mediated via an ERα-dependent mechanism. On the other hand, deletion of ERα had no effect on the ability of polyphenols to decrease oxidative stress and the accumulation of subcutaneous adipose tissue, and to reduce oxidative stress of in the visceral adipose tissue and heart from mice fed with WD.

As expected, WD increased body weight gain more than SD. This is associated with increased fat accumulation, plasma glucose, triglycerides and cholesterol levels. Additionally, it caused oxidative stress in vascular, cardiac, adipose and hepatic tissues.

Here, we showed that polyphenols were not able to significantly decrease weight gain. This finding concurs with a parallel study using the same experimental protocol on mice treated with polyphenols from grape seed (unpublished results) and a previous study on Zucker fatty rats treated with polyphenols extract at the dose of 20 mg/kg/day during 8 weeks (Agouni et al., 2009). Furthermore, 6 weeks of high-fructose diet and simultaneous administration of 50 mg/kg/day or 21 mg/kg/day of the same composition of polyphenols did not modify rat body weight (Al-Awwadi et al., 2004, 2005). Here, we have shown that polyphenols decreased adiposity in particular visceral adipose tissue accumulation, in WT mice. Dietary polyphenols may exert their effects on adipose tissue through one or more signaling and transcriptional pathways potentially including those mediated by NF-κB, AMPK, PPARγ, and PGC-1α (Wang et al., 2014). Among the classes of polyphenols contained in red wine, flavan-3-ols, flavonols and antocyanins have been described to act on adipose tissue. Notably catechins, especially EGCG, have an anti-adipogenic effect via ERK- and CDK-dependent signaling pathway, as well as activation of AMPK and inhibition of lipogenic enzymes (Moon et al., 2007). The flavanols, quercetin and anthocyanins (both at 0.01 mol/kg diet), decreased mesenteric adipose tissue weight in mice fed a high-fat diet for 12 weeks (Hoek-van den Hil et al., 2015). Accordingly, the proposed mechanism underlying the anti-obesity effect exhibited by anthocyanins was mainly mediated by inhibition of preadipocyte proliferation and lipid accumulation during differentiation and reduction of basal lipolysis in 3T3-L1 cells (Kim et al., 2012).

The present study also demonstrated that polyphenols decreased triglyceride plasma levels, which was already reported (Agouni et al., 2009). The extracts used in both studies shared five classes of polyphenols (flavan-3-ols, hydroxycinnamic acids, flavonols, anthocyanins and proanthocyanidins). It can be hypothesized that these classes of polyphenols trigger the decrease of triglyceridemia.

With regards to cardiac function, mice receiving WD did not develop obesity-induced ventricular hypertrophy and diastolic dysfunction as evidenced by echocardiography evaluations. This was in accordance with a previous study showing that mice fed with high-fat diet were unable to recapitulate defects in cardiac function (Brainard et al., 2013). It is possible that the model used in this study represents an early stage of cardiac disease, without the onset of irreversible damage due to hypertrophy or cardiac failure. Although polyphenols did not significantly modify the decreased of cardiac NO^•^ production, it improved WD-induced oxidative stress. Also, in a rat model of metabolic syndrome, polyphenols such as catechin, resveratrol, delphinidin, and gallic acid prevent cardiac ROS overproduction and NADPH overexpression (for review see Le Lay et al., 2014). Moreover, ROS reduction could be explained by the intrinsic capacity of polyphenols to scavenge ROS or alternatively by their ability to modulate expression and activity of antioxidant enzymes.

With regard to vascular function, although polyphenols did not alter responses to Ach and 5-HT, it enhanced NO^•^ production and reduced superoxide anions in aortas leading to increase NO^•^ bioavailability^.^, a notion compatible with our previous data in Zucker fatty rats (Agouni et al., 2009). Indeed, polyphenols were able to enhance NO^•^ bioavailability in aortas from Zucker fatty rats resulting from increased NO^•^ production through enhanced endothelial NOS activity and reduced superoxide anion release via decreased expression of NADPH oxidase membrane sub-unit, Nox-1 (Agouni et al., 2009).

In subcutaneous and visceral adipose tissues, polyphenols were able to completely prevent oxidative stress induced by WD without a significant effect on NO^•^ level. In line with these results, flavonoids such as quercetin, epicatechin and procyanidins, and phenolic acids protect 3T3-L1 preadipocytes from ROS production by enhancing superoxide dismutase antioxidant enzyme gene expression (Marimoutou et al., 2015).

Furthermore, current reports show that polyphenols were able to correct hepatic abnormalities described in obesity, such as impaired NO^•^ production, mitochondrial energy metabolism (Holmstrom et al., 2012) and exaggerated oxidative stress. Emerging evidence suggests that oxidative stress and altered redox balance play a crucial part in the pathogenesis of obesity-related hepatic complications, including steatosis, steatohepatitis and fibrosis, all involving mitochondrial dysfunction. Alterations in the oxidative phosphorylation pathways in liver mitochondria have been reported in various models of obesity with some conflicting results concerning respiratory chain complex (Crescenzo et al., 2008; Vial et al., 2011). It has been shown that dietary supplementation with curcumin diminished mitochondrial dysfunction in the liver of *db/db* mice. This is likely to be due to decreasing lipid peroxidation, resulting in an increase in ATPase activity, restoration of oxygen consumption and NO^•^ synthesis (Soto-Urquieta et al., 2014). It has been reported that high-fat diet affected the activity of all complexes of the respiratory chain in mouse liver (Mingorance et al., 2012). Our results are consistent with the latter at least with the reduced activity of complex II associated with a non-significant decrease of CS activity, supporting either an intrinsic mitochondrial dysfunction or a lower mitochondrial number. Whatever the correct explanation, it is important to note that treatment with polyphenols led to a correction of hepatic complex II activity, as well as NO^•^ and ROS production.

Here, we provide evidence that the alpha isoform of ER participates in the protective *in vivo* effects of polyphenols against WD-induced metabolic defects especially on vascular and liver nitrative stresses and visceral adiposity. It is well known that estrogenic signaling prevents accumulation of visceral fat, increases central sensitivity to leptin, increases the expression of insulin receptors in adipocytes, and decreases the lipogenic activity of lipoprotein lipase in adipose tissue, thus preventing the development of obesity and insulin resistance (Clegg et al., 2006). Accordingly, lowering of adiposity and visceral fat mass accumulation, improvement of vascular function and NO^•^ bioavailability are mediated via an ERα-dependent mechanism. This notion is congruent with previous data showing that polyphenols compounds exert vascular protection and that this effect requires ERα (Chalopin et al., 2010).

**FIGURE 7:**
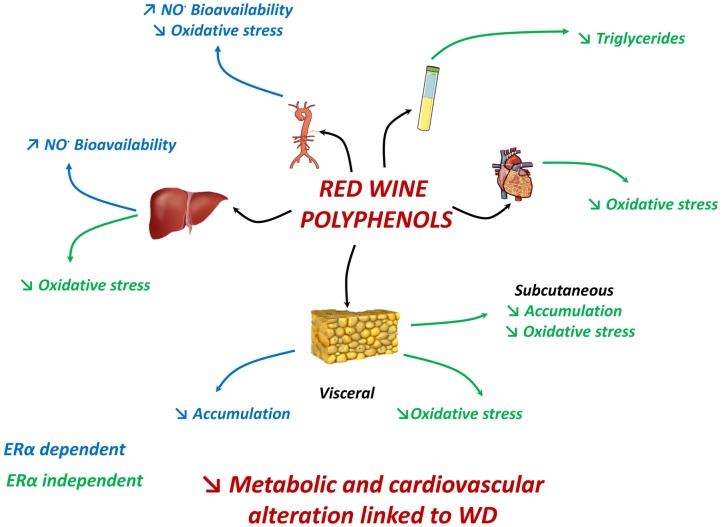
**In WD-fed mice, dietary supplementation with polyphenols reduced aortic ROS, enhanced NO^•^ bioavailability in aorta and liver; and reduced visceral adipose tissue accumulation via an ERα-dependent mechanism.** Polyphenols decreased plasma triglycerides, subcutaneous fat accumulation and ROS in adipose tissues, liver and heart independent of ERα. Hence, effects generated by polyphenols attenuate most features of metabolic dysfunctions partially *via* ERα.

Taken together, these results reveal a scenario in which supplementation of polyphenols during a WD exerts an integrative response able to attenuate most features of metabolic dysfunctions; it also underscores the importance of ERα activation on some of these protective effects including vascular and liver nitrative stresses and visceral adiposity (**Figure 7**). Furthermore, it highlights an additional mechanism of polyphenols to reduce oxidative stress in different tissues involved in obesity-related disorders. Therefore, this study points to the therapeutic potential of red wine extract in metabolic and cardiovascular alterations linked to excessive energy intake.

## Author Contributions

RA conceived the experiments; RA and MM designed the experiments; DL, RS, NC, LV, CJ and LD performed the experiments; DL, RS, NC, CJ, and LD acquired data; DL, RS, NC analyzed data; CD managed financial and administrative tasks; RS, MM, and RA interpreting and discussed the results; RS, MM, and RA wrote and revised the manuscript. All authors read and approved the final manuscript.

## Conflict of Interest Statement

The authors declare that the research was conducted in the absence of any commercial or financial relationships that could be construed as a potential conflict of interest.

## Abbreviations

5-HT: serotonin
A: amplitude
Ach: acetylcholine
AMPK: AMP-activated protein kinase
ANOVA: analysis of variance
A.U.: arbitrary units
bw: body weight
CDK: cyclin-dependent kinase
CMH: 1-hydroxy-3-methoxycarbonyl-2,2,5,5-tetramethylpyrrolidine
COI: cardiac output index
CS: citrate synthase
DETC: Fe2+diethyldithiocarbamate
EF: ejection fraction
EGCG: epigallocatechin-3-gallate
EPR: electron paramagnetic resonance
ERK: extracellular signal-regulated kinase
ERα: estrogen receptor alpha
KO: Knock Out
LVEDD: diastolic left ventricular dimension
LVSED: systolic left ventricular dimension
NF-κB: nuclear factor kappa-light-chain-enhancer of activated B cells
NO^•^: nitric oxide
OVX: ovariectomized
PGC-1α: peroxisome proliferator-activated receptor gamma coactivator-1α
PPARγ: peroxisome proliferator-activated receptors γ
PSS: physiological salt solution
ROS: reactive oxygen species
SD: standard diet
WD: western diet
WT: wild type
